# Selonsertib, a potential drug for liver failure therapy by rescuing the mitochondrial dysfunction of macrophage via ASK1–JNK–DRP1 pathway

**DOI:** 10.1186/s13578-020-00525-w

**Published:** 2021-01-07

**Authors:** Guohua Lou, Aichun Li, Yelei Cen, Qin Yang, Tianbo Zhang, Jinjin Qi, Zhi Chen, Yanning Liu

**Affiliations:** State Key Laboratory for Diagnosis and Treatment of Infectious Diseases, National Clinical Research Center for Infectious Diseases, Collaborative Innovation Center for Diagnosis and Treatment of Infectious Diseases, The First Affiliated Hospital, College of Medicine, Zhejiang University, 79# Qingchun Road, 6A-17, Hangzhou, 310003 China

**Keywords:** Acute liver failure, Mitochondria, Macrophage, ASK1, DRP1

## Abstract

**Background:**

Acute liver failure (ALF) is associated with a high mortality rate, and there are still no effective treatments except liver transplantation and artificial liver therapies. This study aimed to determine the effects, therapeutic window and mechanisms of selonsertib, a selective inhibitor of ASK1, for ALF therapy.

**Results:**

Lipopolysaccharide and d-galactosamine (LPS/GalN) were used to simulate ALF. We found that selonsertib pretreatment significantly ameliorated ALF, as determined by reduced hepatic necrosis and serum alanine aminotransferase, aspartate aminotransferase and inflammatory cytokine levels. However, selonsertib is only effective early after LPS/GalN administration, and the limited therapeutic window is related to the activation and mitochondrial translocation of JNK and DRP1. Further experiments revealed that selonsertib could alleviate LPS-induced mitochondrial damage in macrophages by evaluating the mitochondrial membrane potential and mitochondrial permeability transition pore opening in macrophages. Selonsertib also suppressed the release of inflammatory cytokines from macrophages by reducing DRP1-mediated mitochondrial dysfunction, which was confirmed by using mdivi, a specific DRP1 inhibitor.

**Conclusions:**

Selonsertib protected against LPS/GalN-induced ALF by attenuating JNK-mediated DRP1 mitochondrial translocation and then rescuing mitochondrial damage in macrophages and may have therapeutic potential for early ALF patients.

## Introduction

Acute liver failure (ALF) is a severe consequence of abrupt hepatocyte injury manifested as rapid-onset elevation of aminotransferases, altered mentation, and disturbed coagulation. ALF can be caused by a variety of factors, including paracetamol toxicity, viruses, hepatic ischaemia and autoimmune hepatitis, and drug-induced liver injury from prescription drugs and herbal supplements, and it can evolve over days or weeks to a lethal outcome [1]. Liver transplantation and artificial liver therapies are presented as the main clinical treatments for ALF. However, the shortage of available donor livers limits the widespread clinical application of liver transplantation, and multiple postoperative complications also limit the efficacy of artificial liver therapy against ALF [2]. Thus, there is an urgent medical need to identify novel therapies for ALF.

Apoptosis signal-regulating kinase 1 (ASK1) is a ubiquitously expressed apical mitogen-activated kinase kinase kinase (MAP3K) that is activated by various types of pathological stimuli, including neurodegenerative disorders, inflammatory diseases and cancer [3–5]. Recently, several investigations have shown that ASK1 is overactivated in ALF and liver injury, such as APAP-induced ALF, CCl_4_-induced liver injury, and hepatic ischaemia/reperfusion injury, suggesting that inactivation of ASK1 might be a potential strategy for ALF therapy [6, 7]. Selonsertib, a selective inhibitor of ASK1, has been reported as an effective treatment for non-alcoholic steatohepatitis (NASH) and multidrug resistance (MDR) in various types of cancer in human patients by reducing hepatic steatosis, inflammation and fibrosis or reversing ATP-binding cassette transporter-mediated MDR [8, 9]. Its phase III clinical trial on NASH was initiated by the U.S. Food and Drug Administration [10].

Lipopolysaccharide and d-galactosamine (LPS/GalN) are widely used to simulate ALF [11]. The present study aimed to investigate the effects, underlying mechanisms, and therapeutic window of selonsertib in LPS/GalN-induced ALF.

## Methods

### Animals

C57BL/6 mice (aged 5–6 weeks) were purchased from Nanjing BioMedical Research Institute of Nanjing University (Nanjing, China). Animals were housed in a controlled environment with a 12-h light/dark cycle and free access to food and water. All experimental protocols were approved by the Institutional Animal Care and Use Committee of the First Affiliated Hospital of Zhejiang University.

### ALF mouse model and treatment

C57BL/6J mice were intraperitoneally injected with LPS (10 μg/kg, Sigma-Aldrich, St Louis, MO, USA) and D-GalN (400 mg/kg, Sigma-Aldrich) to establish a mouse model of LPS/GalN-induced ALF. Selonsertib (15, 30, and 60 mg/kg, MCE) was administered via i.p. injection 30 min prior to LPS/GalN injection or at 0.5, 1, 2, and 4 h after LPS/GalN injection. A JNK activator, anisomycin (20 mg/kg, MCE), was administered via i.p. injection combined with selonsertib to further investigate the role of the JNK pathway in mediating the protective effect of selonsertib. For another therapy, mdivi (30 mg/kg, MCE) was administered via *i.p.* injection 30 min prior to LPS/GalN injection. The control group was administered vehicle (n = 6). At 0.5, 1, 2, 4, and 6 h after LPS/GalN injection, the mice were sacrificed, and serum and liver samples were collected to assess the extent of liver injury. Serum was evaluated for biochemical parameters. The liver samples were evaluated for histochemistry and Western blot analysis.

### Liver histological and serum biochemical analysis

Liver tissues were processed for paraffin embedding and sectioned into 4 μm sections. The sections were then routinely stained with haematoxylin and eosin (H&E staining). The serum levels of alanine aminotransferase aspartate (ALT) and aspartate aminotransferase (AST) were measured by using FUJI DRI-CHEM Slide GFP/ALT-PIII and GOT/AST-PIII, respectively, according to the manufacturer's instructions with DRI-CHEM 4000ie (FUJIFILM). Total bilirubin (TBiL) was measured using standard clinical chemistry techniques (Integra II; Roche, UK).

### Cell culture and treatment

The mouse macrophage cell line RAW264.7 was cultured in 1640 medium (Thermo Fisher Scientific, Inc.) containing 10% foetal bovine serum (Thermo Fisher Scientific, Inc.), 100 U/ml penicillin, and 100 μg/ml streptomycin (Thermo Fisher Scientific, Inc.) and maintained at 37 °C in a humidified atmosphere containing 5% CO_2_. The cells were preincubated with selonsertib (5 µM) or mdivi (10 µM) for 6 h and then incubated with LPS (500 ng/ml) for 4 h. The supernatant and cell samples were then collected for further analysis.

### Cytokine detection by ELISA

The cytokine levels in supernatants from cell cultures were quantified with an enzyme-linked immunosorbent assay (ELISA) with commercial mouse tumour necrosis factor-α (TNF-α) and interleukin-1α (IL-1α, MultiSciences, China) ELISA kits according to the manufacturer’s instructions.

### Cytometric Bead Array (CBA) analysis

Cytokine levels in the serum samples were detected by a BD LSR Fortessa cytometer (Becton Dickinson Holdings Pte Ltd, Singapore) using a commercially available BD™ Cytometric Bead Array (CBA) Mouse Inflammation Kit (BD Biosciences, USA) following the manufacturer’s instructions [12]. In brief, cytokine standards were prepared using a vial of lyophilized mouse inflammatory cytokines, including IL-6, IL-10, monocyte chemoattractant protein-1 (MCP-1), interferon-γ (IFN-γ), TNF-α, and IL-12p70, and assay diluent by serial dilutions. Capture beads were added to each tube containing samples, standards, and negative controls and incubated for 30 min at room temperature in the absence of light. The flow cytometer was calibrated using cytometer setup beads, and the assay was performed.

### Isolation of hepatic macrophages

Hepatic macrophages were isolated from different groups of mice using the collagenase liver perfusion system. Briefly, the liver was perfused with digestion buffer containing collagenase for 5 min and then excised and shaken in RPMI 1640 medium with 0.1% type IV collagenase for 15 min. Perfused liver tissue was gently dispersed in RPMI 1640 medium containing 10% FBS and filtered through a 70 μm mesh. After centrifugation at 50×*g* for 5 min at 4 °C, the hepatocyte fraction was discarded. Nonparenchymal cell fractions were carefully layered on top of Percoll gradients composed of 25% and 50% Percoll layers. After centrifugation at 800×*g* for 30 min at 4 °C without braking, the hepatic macrophage fraction was isolated between the two different Percoll gradient layers. Then, the isolated cells were seeded into plates. After 30 min, the unattached cells were removed, and the remaining attached cells were collected as primary hepatic macrophages.

### Mitochondrial permeability transition pore (mPTP) opening detection and mitochondrial membrane potential observation

The mitochondrial membrane potential (Δψm) was observed by staining with the JC-1 probe (Beyotime Biotechnology, Jiangsu, China) according to the manufacturer’s instructions. After JC-1 incubation for 20 min at 37 °C, monomeric green fluorescence emissions and aggregate red fluorescence intensities in cells were observed by two-photon confocal laser scanning microscopy (Lei TCS SPB-MaiTai MP). mPTP opening was evaluated using the mPTP Assay Kit (Beyotime Biotechnology) according to the manufacturer’s instructions and observed with the above microscopy. A decrease in green fluorescence indicated increased opening of the mPTP.

### Determination of mitochondrial oxidative stress

Cells were pelleted by centrifugation, resuspended and incubated in prewarmed growth medium containing 250 nM MitoSOX™ Red Mitochondrial Superoxide Indicator (Thermo Fisher Scientific, Inc.) for 30 min at 37 °C in a 5% CO_2_ atmosphere. After incubation, the cells were washed with PBS, and the fluorescence was then assessed by fluorescence microscopy.

### Mitochondria isolation

Mitochondria from macrophages and liver tissues were isolated by using the Cell Mitochondria Isolation Kit (Beyotime Biotechnology) and Tissue Mitochondria Isolation Kit (Beyotime Biotechnology), respectively, according to the manufacturer’s instructions. The protein expression levels in the isolated mitochondria were then examined by Western blot analysis.

### Western blot analysis

To determine protein expression levels, whole-cell, cell mitochondrial cytoplasmic fractions, tissue extracts, and tissue mitochondrial fractions were lysed with RIPA peptide lysis buffer (Beyotime Biotechnology) containing 1% protease inhibitors (Pierce). Samples were quantified using a Pierce BCA Protein Assay Kit according to the manufacturer’s instructions, and 30 μg protein was resolved by SDS-PAGE and transferred to PVDF membranes. Membranes were blocked in 5% non-fat milk and incubated overnight at 4 °C with primary antibodies, including anti-ASK1 (1:1000; Cell Signaling Technology), anti-p-ASK1 (1:1000; Cell Signaling Technology), anti-JNK (1:1000; Cell Signaling Technology), anti-p-JNK (1:1000; Cell Signaling Technology), anti-p38 (1:1000; Cell Signaling Technology), anti-p-p38 (1:1000; Cell Signaling Technology), anti-dynamin-related protein 1 (DRP1, 1:1000; Abcam), anti-p-DRP1 (1:1000; Cell Signaling Technology), anti-OPA1 (1:1000; Abcam), anti-mitofusin 1 (MFN1, 1:1000; Abcam), anti-mitofusin 2 (MFN2, 1:1000; Abcam), anti-mitochondrial fission protein (MFF, 1:1000; Abcam), and anti-mitochondrial fission protein 1 (FIS1, Proteintech), anti-voltage-dependent anion channel 1 (VDAC1, 1:1000; Abcam), and anti-GAPDH (1:3000; Cell Signaling Technology). The membranes were then probed with HRP-tagged secondary antibodies at room temperature for 1 h. The protein bands were visualized using an enhanced chemiluminescence system and detected in the ChemiScope Western Blot Imaging System (Clinx Science Instruments Co., Ltd.). ImageJ software was used for greyscale value assay.

### Statistics

All data are presented as the mean ± SD. Comparisons were made between two groups using independent samples t-tests or between three or more groups using one-way analysis of variance (ANOVA) with Tukey’s post hoc test. *P* < 0.05 was considered significant.

## Results

### Selonsertib protects against LPS/GalN-induced ALF by inhibiting the activation of the JNK pathway

**Fig. 1:**
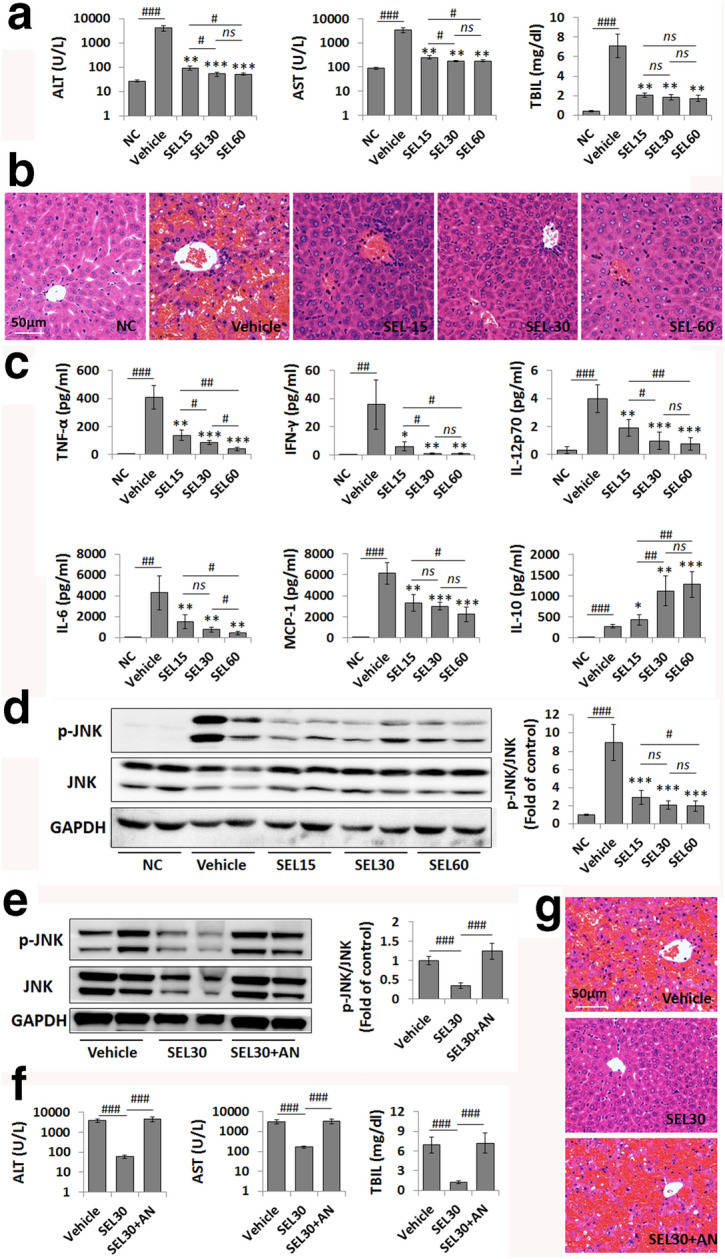
Selonsertib administration ameliorates LPS/GalN-induced liver failure. **a** Serum levels of ALT, AST, and TBIL were elevated by LPS/GalN injection (vehicle group) and decreased by selonsertib treatment in a dose-dependent manner. **b** Pathological changes in the liver tissue were shown using H&E staining. **c** The serum levels of inflammatory cytokines were determined by CBA cytokine analysis. **d**, **e** Western blot analysis and grey value assay of p-JNK and JNK expression levels in murine liver samples from different groups (2 from 6 samples are shown in the blots). **f**, **f** Serum levels of ALT, AST, and TBIL (**f**) and H&E staining (**g**) in LPS/GalN-injected mice treated with vehicle, selonsertib, or selonsertib combined with anisomycin. Data are presented as the mean ± SD (n = 6). *P < 0.05 vs. vehicle; **P < 0.01 vs. vehicle; ***P < 0.001 vs. vehicle; ^#^*P* < 0.05; ^##^*P* < 0.01; ^###^*P* < 0.001; *ns* not significant, *NC* normal control, *SEL15* selonsertib (15 mg/kg), *SEL30* selonsertib (30 mg/kg), *SEL60* selonsertib (60 mg/kg), *AN* anisomycin (20 mg/kg)

To investigate the effect of selonsertib on LPS/GalN-induced ALF, mice were treated with 15, 30, or 60 mg/kg selonsertib 30 min prior to administration of LPS/GalN and then examined 6 h later. As shown in Fig. 1a, b, LPS/GalN induced significant liver injury, as indicated by dramatically elevated serum ALT, AST, and TBiL levels accompanied by massive hepatic necrosis, while pretreatment with all three dosages of selonsertib resulted in a remarkable reduction in serum ALT, AST, and TBiL levels (Fig. 1a) and less hepatic necrosis (Fig. 1b). CBA cytokine analysis also showed that selonsertib pretreatment could significantly reduce the elevated serum levels of inflammatory cytokines and chemokines, such as TNF-α, IFN-γ, IL-12p70, IL-6, and MCP-1, whereas selonsertib pretreatment could enhance the serum level of IL-10, an anti-inflammatory cytokine, in LPS/GalN-administered mice, especially at dosages of 30 and 60 mg/kg (Fig. 1c). These data indicate that selonsertib can effectively prevent LPS/GalN-induced ALF.

As selonsertib is a specific ASK1 inhibitor, we then determined the protective mechanism of selonsertib in this model by focusing on the ASK1-mediated signalling pathway. JNK activation has been implicated in a variety of liver injuries, and ASK1 is suggested to function upstream of the JNK pathway [3, 13]. As expected, LPS/GalN administration induced a remarkable elevation in phosphorylated JNK (p-JNK) levels in liver tissues, while selonsertib pretreatment markedly reduced the phosphorylation of JNK (Fig. 1d). Quantitative densitometric analysis also confirmed the changes in p-JNK/total JNK levels by pretreatment with all three doses of selonsertib. In contrast, combined treatment with a JNK activator, anisomycin [14], the reduced hepatic JNK activation induced by selonsertib pretreatment was completely reversed, and the protective effects of selonsertib on ALF were also markedly abolished (Fig. 1e–g). These results confirmed that the inhibition of ASK1-mediated JNK activation by selonsertib may contribute to the prevention of LPS/GalN-induced ALF.

### Selonsertib efficacy is related to JNK mitochondrial translocation

**Fig. 2:**
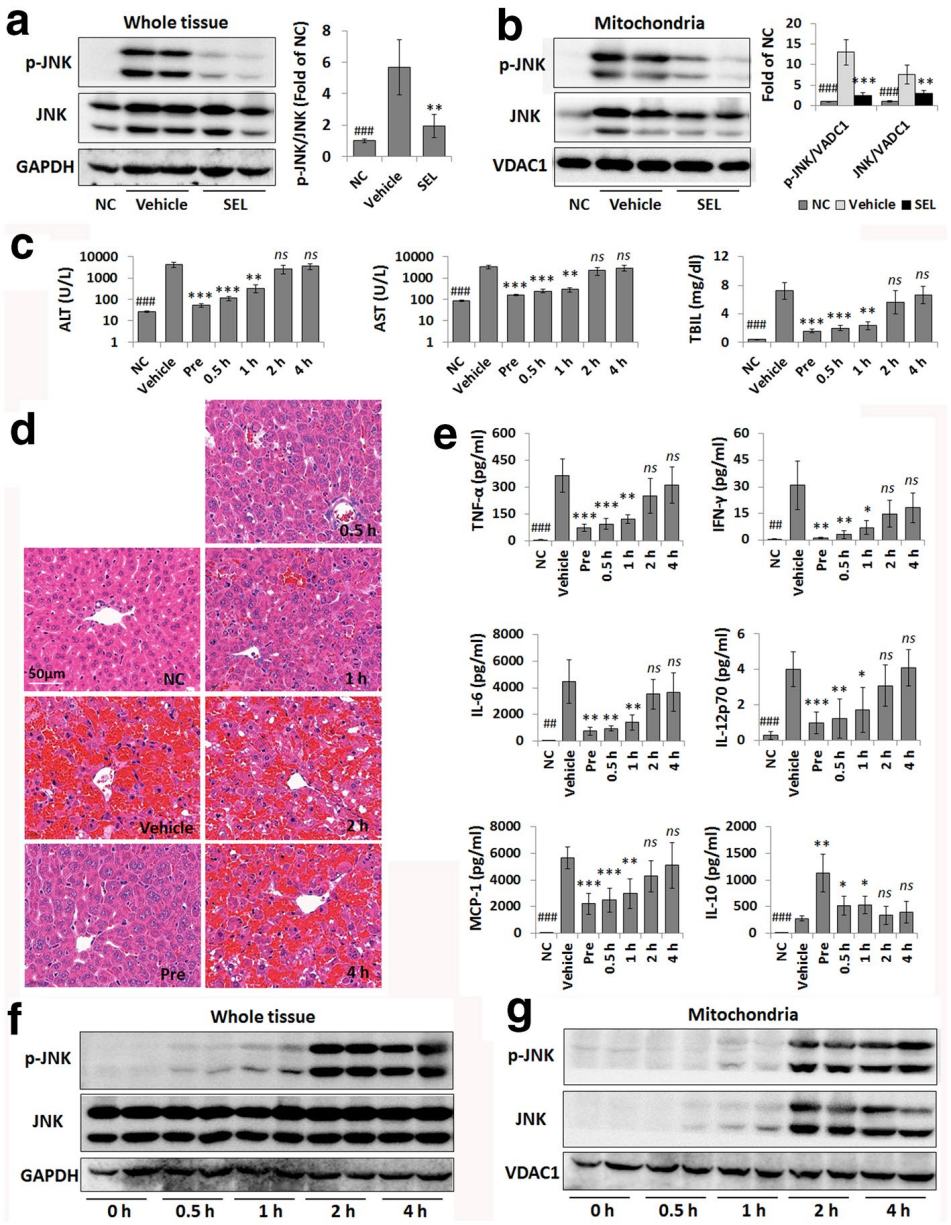
The therapeutic efficacy of selonsertib is related to JNK mitochondrial translocation. **a**, **b** Western blot analysis and grey value assay of p-JNK and JNK levels in murine whole liver samples (**a**) and the mitochondrial fraction of liver samples (**b**); 1 or 2 from 6 samples are shown in the blots. **c** Serum levels of ALT, AST, and TBIL in different groups with selonsertib pretreatment (Pre) or treatment at 0.5 h, 1 h, 2 h, and 4 h after LPS/GalN injection. **d** Pathological changes in the liver tissue were shown by H&E staining. **e** The serum levels of inflammatory cytokines and chemokines were determined by CBA cytokine analysis. **f**, **g** Western blot analysis of p-JNK and JNK levels in murine whole liver samples (**f**) and the mitochondrial fraction of liver samples (**g**) at initial (0 h) and 0.5 h, 1 h, 2 h, and 4 h after LPS/GalN injection (2 from 6 samples are shown in the blots). Data are presented as the mean ± SD (n = 6). *P < 0.05 vs. vehicle; **P < 0.01 vs. vehicle; ***P < 0.001 vs. vehicle; ^##^*P* < 0.01 vs. vehicle; ^###^*P* < 0.001 vs. vehicle; *ns* not significant vs*.* vehicle, *NC* normal control, *SEL* selonsertib (30 mg/kg)

Since sustained JNK activation and translocation to mitochondria have been implicated in liver injury [13, 15], we then assessed the effect of selonsertib on the activation and mitochondrial translocation of JNK. As shown in Fig. 2a, b, the LPS/GalN-induced elevation in p-JNK (Fig. 2a) was accompanied by increases in total JNK and p-JNK in mitochondria (Fig. 2b), and this was prevented by administration of selonsertib.

We then analysed the therapeutic window of selonsertib in this model. Mice treated with selonsertib 0.5 h or 1 h after LPS/GalN also showed significant protection against liver injury when compared to vehicle-treated mice but when selonsertib administration was delayed to 2 h or 4 h post LPS/GalN, no protection against liver injury were evident as determined by serum ALT, AST and TBiL levels (Fig. 2c), H&E staining (Fig. 2d), and CBA cytokine analysis (Fig. 2e). We further investigated whether the limited therapeutic window of selonsertib on ALF was related to aberrant JNK activation. Limited LPS/GalN-induced JNK activation was observed as early as 0.5 h and 1 h with almost no JNK and p-JNK translocation to the mitochondria. However, by 2 h, massive JNK activation and extensive mitochondrial translocation had occurred (Fig. 2f, g). The above data suggested that the protective effect of selonsertib on LPS/GalN-induced ALF was dependent on the suppression of early JNK activation and mitochondrial translocation.

### Selonsertib suppresses DRP1 mitochondrial translocation and rectifies abnormal mitochondrial dynamics

**Fig. 3:**
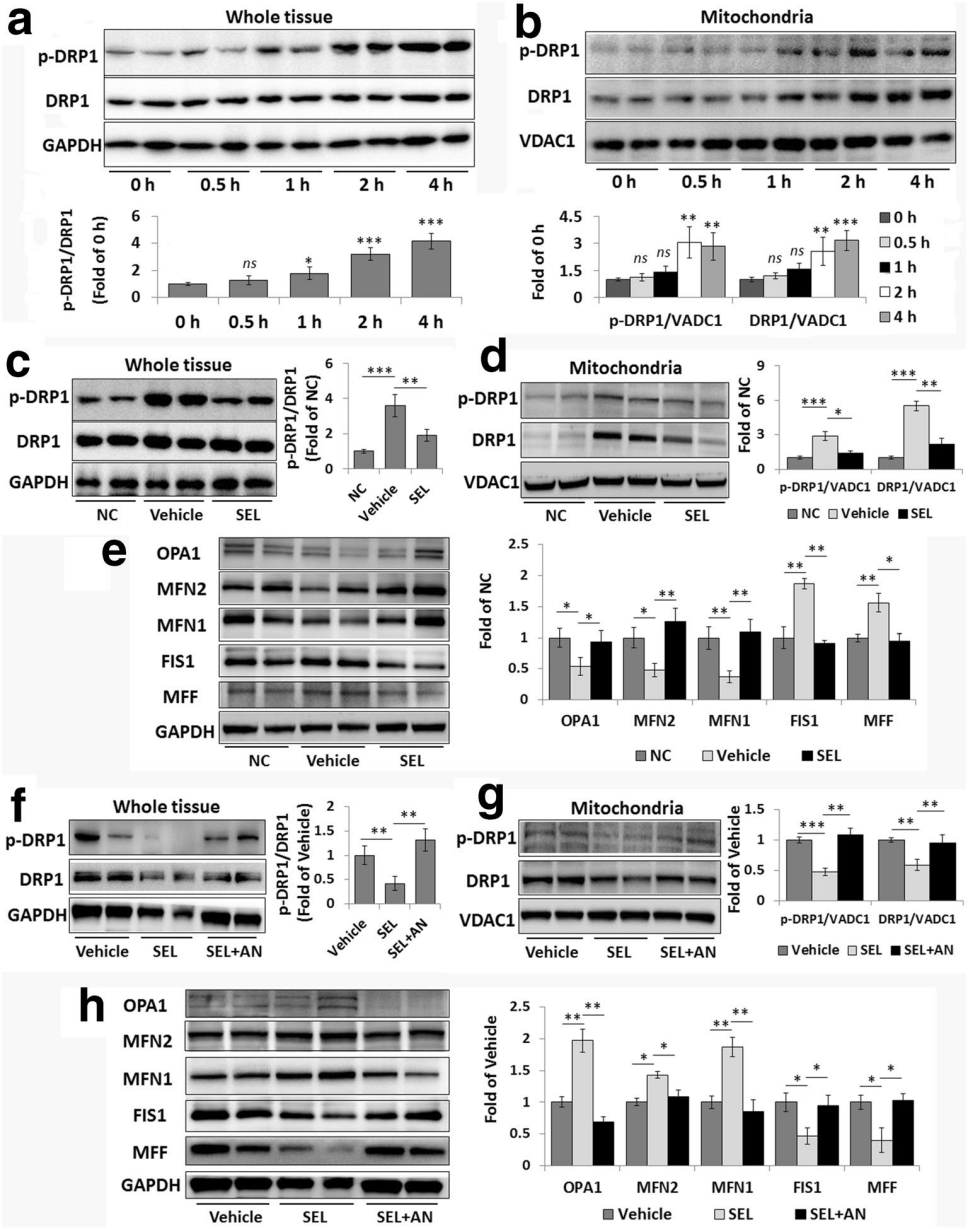
Selonsertib reverses JNK-mediated DRP1 mitochondrial translocation and abnormal mitochondrial dynamics. **a**, **b** Western blot analysis and grey value assay of p-DRP1 and DRP1 levels in murine whole liver samples (**a**) and the mitochondrial fraction of liver samples (**b**) at initial (0 h) and 0.5 h, 1 h, 2 h, and 4 h after LPS/GalN injection. **c**, **d** Western blot analysis and grey value assay of p-DRP1 and DRP1 levels in murine whole liver samples (**c**) and the mitochondrial fraction of liver samples (**d**) from different groups. **e** Western blot analysis and grey value assay of the expression levels of mitochondrial dynamics-related proteins in murine liver samples. **f**–**h** Anisomycin was used to further confirm the role of JNK in mediating the effects of selonsertib on DRP1 activation and mitochondrial dynamics. Western blot analysis and grey value assay of DRP1 activation and mitochondrial translocation in murine whole liver samples (**f**) and the mitochondrial fraction of liver samples (**g**) and the expression levels of mitochondrial dynamics-related proteins in murine liver samples (**h**). Two from 6 samples were shown in the blots. Data are presented as the mean ± SD (n = 6). *P < 0.05; **P < 0.01; ***P < 0.001; *NC* normal control, *SEL* selonsertib (30 mg/kg), *AN* anisomycin (20 mg/kg)

As DRP1 translocation to mitochondria has been suggested to be downstream of JNK activation and mediate mitochondrial fission [16, 17], we then investigated the effect of selonsertib on JNK-mediated DRP1 activation and mitochondrial translocation in the whole and mitochondrial fractions of liver tissues from mice following 0.5, 1, 2, and 4 h LPS/GalN exposure. As shown in Fig. 3a, LPS/GalN administration induced a gradual elevation in DRP1 phosphorylation at Ser616 (p-DRP1) in whole tissue samples, and a dramatic increase in p-DRP1 was detected at 2 h after LPS/GalN injection. Further analyses of the levels of DRP1 and p-DRP1 proteins in the mitochondrial fraction showed that LPS/GalN injection promoted the translocation of both DRP1 and p-DRP1 to mitochondria, especially 2 h after LPS/GalN injection (Fig. 3b). Interestingly, selonsertib pretreatment effectively suppressed the elevation of DRP1 phosphorylation and its translocation to mitochondria by LPS/GalN induction (Fig. 3c, d).

As altered DRP1 activity is known to result in abnormal mitochondrial dynamics [18], we then investigated the effect of selonsertib on mitochondrial dynamics by determining the expression of mitochondrial fission- and fusion-related proteins. Compared with those in the normal control (NC) group, the expression of fission-related proteins FIS1 and MFF was significantly upregulated, whereas the expression of the fusion-related proteins MFN1, MFN2, and OPA1 was concurrently significantly decreased in the liver tissue from LPS/GalN-injected mice (Fig. 3e). However, the dysregulated expression of mitochondrial dynamics-related proteins was markedly rescued by selonsertib pretreatment.

We further used anisomycin to confirm whether the effects of selonsertib-reduced DRP1 activation and mitochondrial translocation are specifically mediated by JNK inhibition. When anisomycin was used together with selonsertib, DRP1 phosphorylation and mitochondrial translocation and the expression of mitochondrial dynamics-related proteins in the liver were significantly affected, suggesting that anisomycin can interfere with selonsertib-rectified mitochondrial dynamics (Fig. 3f–h). These data suggest that selonsertib may prevent ALF by modulating mitochondrial dynamics by suppressing JNK-mediated DRP1 mitochondrial translocation.

### Selonsertib prevents mitochondrial damage to macrophages in ALF by suppressing DRP1 activation

**Fig. 4:**
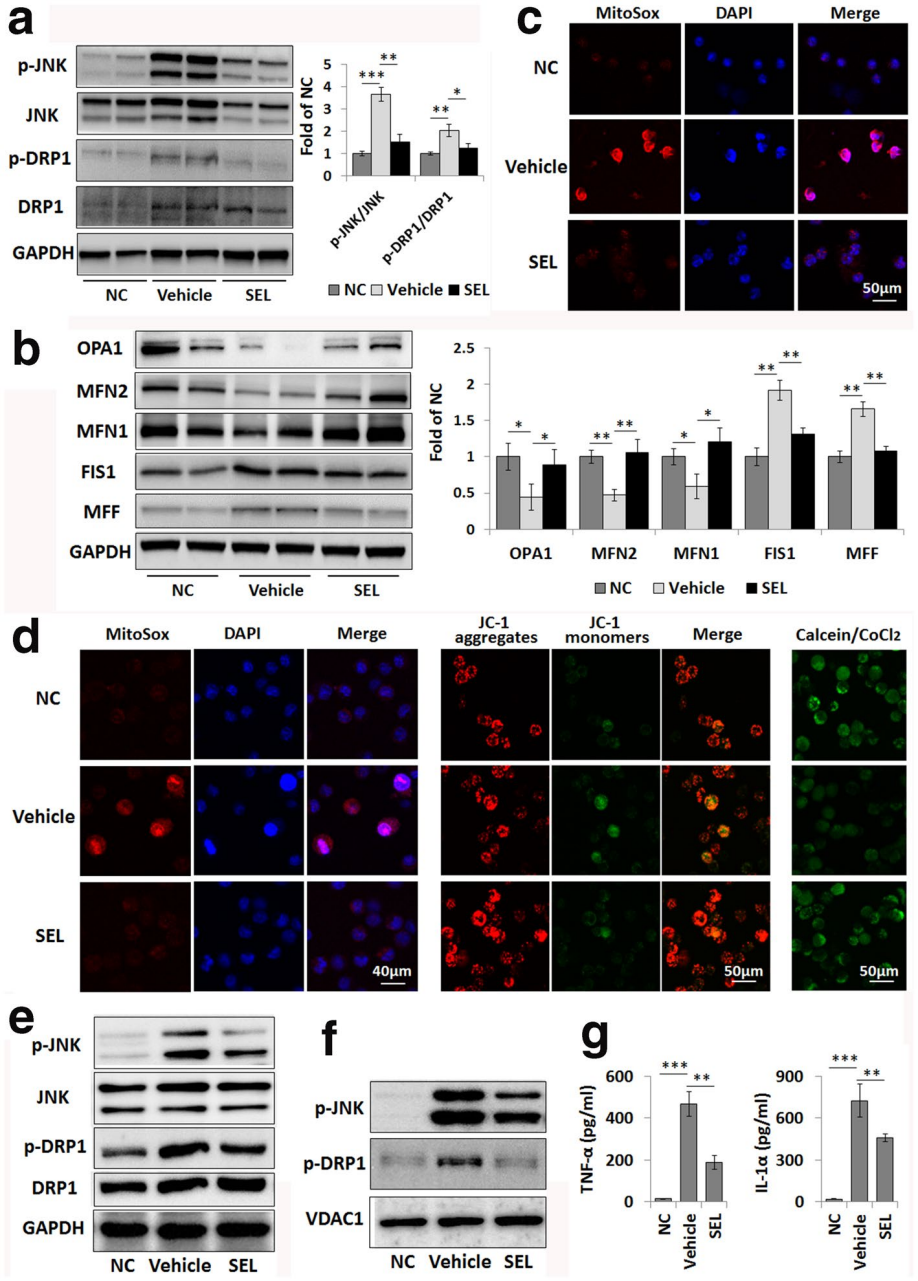
Selonsertib prevents LPS-primed JNK and DRP1 mitochondrial translocation and mitochondrial damage in macrophages. **a**, **b** Western blot analysis of p-JNK, JNK, p-DRP1 and DRP1 levels (**a**) and mitochondrial dynamics-related protein levels in primary hepatic macrophages isolated from different groups of LPS/GalN-injected mice. Two from 6 samples were shown in the blots. **c** Determination of mitochondrial oxidative stress in primary hepatic macrophages by using MitoSox. **d** Mitochondrial oxidative stress, mitochondrial membrane potential (Δψm), and mitochondrial permeability transition pore (mPTP) opening in normal cultured (NC) and LPS-primed RAW264.7 cells are shown by MitoSox (left), JC-1 (middle) and calcein staining (right), respectively. **e**, **f** Western blot analysis of p-JNK, JNK, p-DRP1 and DRP1 levels in the whole cell (**e**) and the mitochondrial fraction (**f**) of RAW264.7 cells. **g** TNF-α and IL-1α secretion levels from RAW264.7 cells after LPS exposure were measured by ELISA. Data are presented as the mean ± SD (n = 3). **P < 0.01; ***P < 0.001; *NC* normal control, *SEL* selonsertib (30 mg/kg for in vivo; 5 µM for in vitro)

Macrophages play a pivotal role in modulating the hepatic immune microenvironment and inflammatory response in ALF [19]. As previous studies showed that LPS exposure usually induced mitochondrial dysfunction in macrophages [20, 21], we further investigated the effect of selonsertib in protecting the mitochondrial function and dynamics of hepatic macrophages. As shown in Fig. 4a, the activation of JNK and DRP1 in primary hepatic macrophages from LPS/GalN-injected mice pretreated with selonsertib was significantly alleviated compared with that in mice treated with vehicle. At the same time, the dysregulated expression of mitochondrial dynamics-related proteins in hepatic macrophages from ALF mice was also markedly rescued by selonsertib pretreatment (Fig. 4b). Additionally, the marked elevation of mitochondrial oxidative stress, as indicated by MitoSox, in the hepatic macrophages from LPS/GalN-injected mice was decreased in the group with selonsertib pretreatment compared to those treated with vehicle (Fig. 4c).

We also confirmed the role of selonsertib in protecting mitochondrial function in the LPS-primed mouse macrophage cell line RAW264.7. As expected, LPS exposure induced a marked increase in mitochondrial oxidative stress in macrophages, while selonsertib treatment significantly weakened the red fluorescence intensity in LPS-primed macrophages (Fig. 4d-left). In addition, LPS priming caused progressive opening of the mPTP, as inferred from the apparent decrease in the fluorescent probe calcein, which had been preloaded into mitochondria (Fig. 4a-right). LPS priming also decreased the mitochondrial membrane potential (Δψm), as indicated by the increased conversion of red fluorescence to green fluorescence of JC-1 dye (Fig. 4d-middle). However, selonsertib pretreatment effectively prevented LPS-induced mPTP opening and Δψm dissipation in macrophages, as shown by mitochondrial accumulation of calcein fluorescence and reduced the ratio of red to green fluorescence of JC-1 dye in macrophages (Fig. 4d). Moreover, LPS exposure also induced the activation and mitochondrial translocation of JNK and DRP1 in macrophages, as we observed in the liver tissue of LPS/GalN-induced ALF, while selonsertib pretreatment effectively suppressed these activation and mitochondrial translocation by LPS priming (Fig. 4e, f). Meanwhile, LPS stimulation induced significant release of inflammatory cytokines, such as TNF-α and IL-1α, whereas selonsertib treatment alleviated the release of these cytokines (Fig. 4g).

**Fig. 5:**
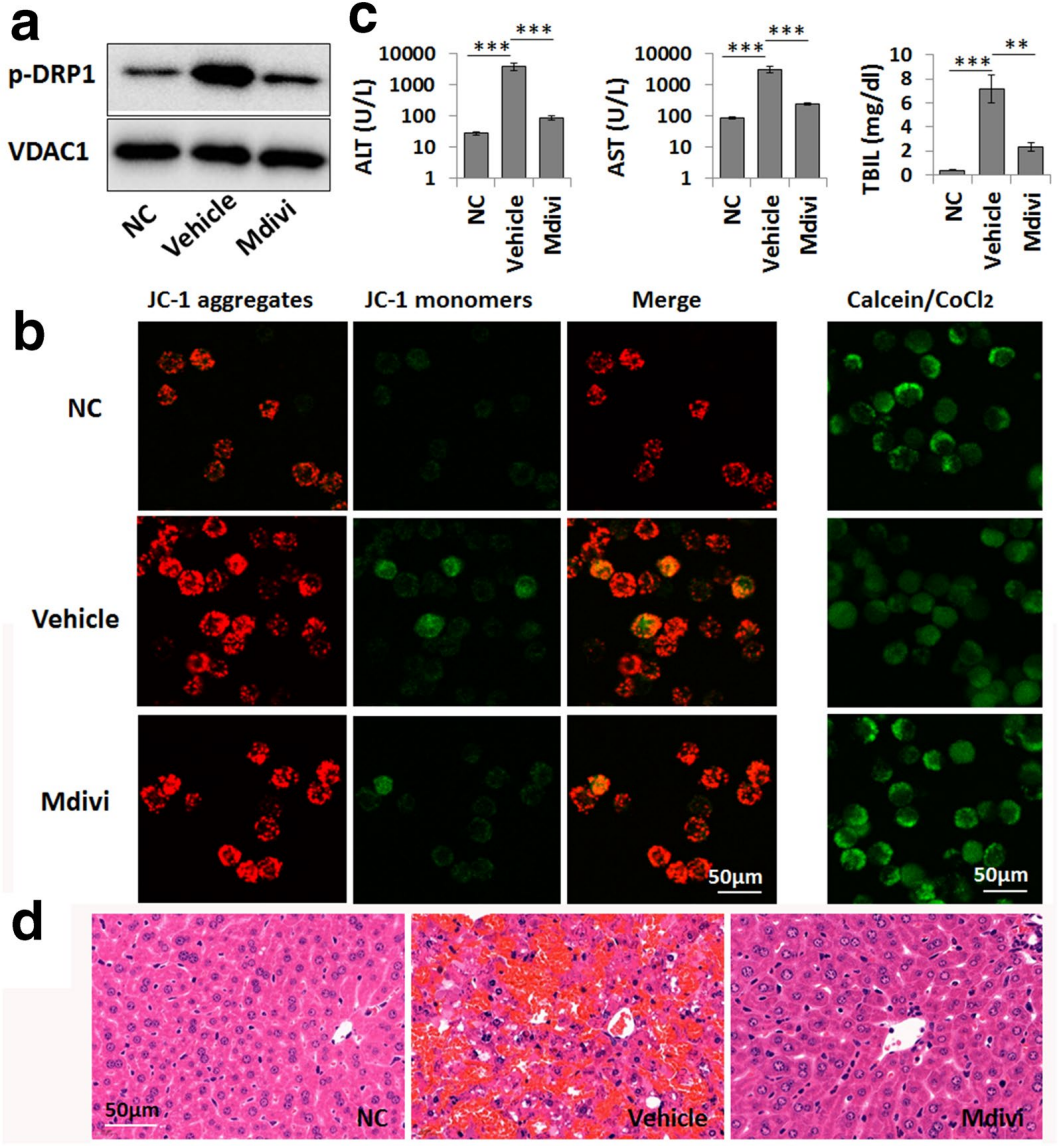
DRP1 mediates mitochondrial damage in macrophages in LPS/GalN-induced liver failure. **a** Western blot analysis of p-DRP1 levels in the mitochondrial fraction of normal cultured (NC) and LPS-primed macrophages. Mdivi (10 µM) was used to inhibit DRP1 activity. **b** The mitochondrial membrane potential (Δψm) and mitochondrial permeability transition pore (mPTP) opening in normal cultured (NC) and LPS-primed macrophages are shown by JC-1 (left) and calcein staining (right), respectively. **c** Serum levels of ALT, AST and TBIL in the normal control group (NC), vehicle-treated ALF group (Vehicle), and mdivi-treated group (Mdivi, 30 mg/kg). Data are presented as the mean ± SD (n = 6). **P < 0.01; ***P < 0.001. **d** Pathological changes in the liver tissue were shown by H&E staining

By using mdivi, a DRP1 inhibitor, we further found that the inhibition of DRP1 activation could effectively inhibit mitochondrial translocation and then alleviate LPS-primed abnormal mitochondrial dynamics and mitochondrial dysfunction, which were indicated by the alleviation of the abnormal expression of mitochondrial dynamics-related proteins and the reduction in mPTP opening and Δψm dissipation in LPS-primed macrophages (Fig. 5a, b). We further confirmed the protective effects of mdivi on LPS/GalN-induced ALF by observing reduced serum ALT, AST, and TBiL levels and reduced hepatic necrosis in these mice after mdivi pretreatment (Fig. 5c, d). Thus, JNK-mediated DRP1 mitochondrial translocation might be an important link that mediates the protective effect of selonsertib on ALF and mitochondrial damage.

## Discussion

**Fig. 6:**
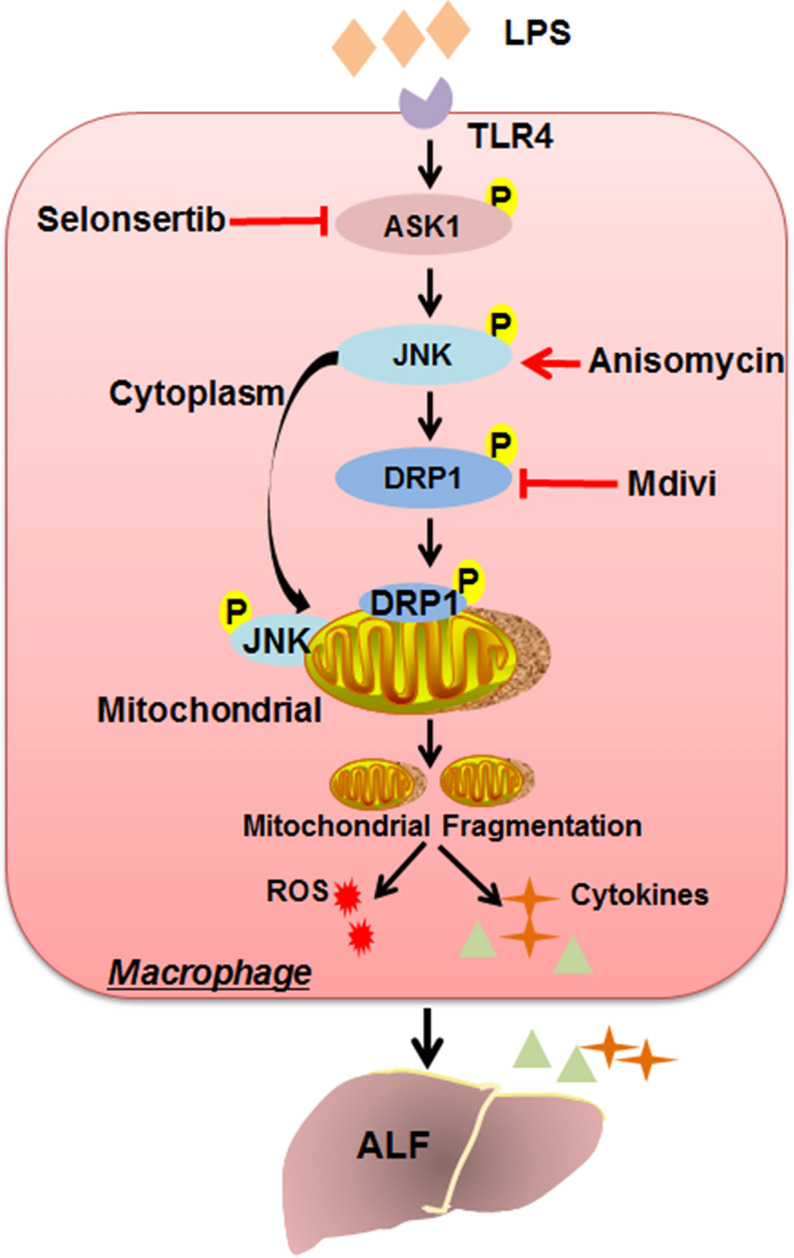
Schematic depicting the protective mechanism of selonsertib against mitochondrial damage. In LPS/GalN-induced ALF, excessive ASK1 phosphorylation induces JNK-mediated DRP1 activation and mitochondrial translocation in macrophages, which in turn promotes excessive mitochondrial fission. Enhanced mitochondrial fragmentation then leads to mitochondrial damage, an increase in mitochondrial oxidative stress, and the overproduction of inflammatory cytokines by macrophages. Selonsertib, a selective inhibitor of ASK1, protects against ALF by re-establishing the mitochondrial fusion-fission balance through suppressing the ASK1–JNK–DRP1 pathway in macrophages, rescuing mitochondrial damage and relieving inflammatory injury

ALF, also known as fulminant hepatic failure, is a life-threatening clinical syndrome induced by massive hepatic necrosis. Its medical management is associated with high rates of morbidity and mortality, and there are still no effective treatments except liver transplantation and artificial liver therapies [1]. Thus, it is important to identify a potential drug, further clarify its treatment mechanism and determine its therapeutic window for ALF. In this study, we showed that selonsertib, a selective inhibitor of ASK1 that is currently in a phase III clinical trial on NASH [22], could effectively protect against LPS/GalN-induced ALF by inhibiting the activation of the hepatic ASK1–JNK–DRP1 pathway and alleviating mitochondrial damage in macrophages (Fig. 6). Unfortunately, selonsertib is only effective early after LPS/GalN administration, and the limited therapeutic window may be related to the time point of JNK and DRP1 mitochondrial translocation.

ASK1, a mammalian MAP3K, is activated in response to various cytotoxic stresses, including serum withdrawal, ROS, tumour necrosis factor, microtubule interfering agents, and cancer chemotherapeutic agents, and it has been implicated in a variety of cellular functions, including cell survival and inflammatory response [5, 23, 24]. Activated ASK1 then activates downstream kinases such as the JNK and p38 pathways, resulting in cell apoptosis and inflammatory cytokine expression [3, 25]. Recently, several reports have shown that ASK1 is overactivated in a variety of liver diseases, including drug- and toxin-induced ALF [6, 26, 27]. In this study, we found that ASK1 was significantly activated in the liver tissues of LPS/GalN-induced ALF (Additional file 1: Figure S1A). Thus, as a specific inhibitor of ASK1, selonsertib might be a potential drug for ALF therapy. As we expected, pretreatment with selonsertib remarkably lessened hepatic necrosis and reduced serum levels of ALT, AST, TBiL, and inflammatory cytokines and chemokines in a dose-dependent manner. In addition, selonsertib pretreatment suppressed the activation of JNK (Fig. 1) and p38 (Additional file 1: Figure S1A) in live tissue from the ALF mouse model. Other studies also supported the above findings, as ASK1 inhibition protected against acetaminophen-induced liver injury and high-fat diet-induced hepatic steatosis by attenuating JNK and p38 activation [6, 28]. Unfortunately, further experiments showed that selonsertib is only effective early after LPS/GalN administration (within 1 h in the model) and loses its efficacy relatively rapidly after that time. The limited therapeutic window of selonsertib on ALF may be due to the time point of JNK activation and mitochondrial translocation, as limited LPS/GalN-induced JNK activation and mitochondrial translocation were observed as early as 1 h, whereas massive JNK activation and extensive mitochondrial translocation occurred 2 h later. However, there was no obvious regularity in the activation of hepatic p38 after LPS/GalN exposure (Additional file 1: Figure S1B), suggesting that in this model, ASK1-mediated JNK activation and mitochondrial translocation might be the key factors determining selonsertib therapeutic efficiency. A previous study also showed that JNK, but not p38, contributed to ASK1-mediated cellular damage in direct APAP-induced hepatocyte injury [26].

The primary pathophysiological event in LPS/GalN-induced ALF is the release of proinflammatory cytokines derived from macrophages [11, 29]. Emerging evidence now indicates that mitochondrial dysfunction is a possible mechanism of LPS/GalN-induced ALF, which causes abnormal mitochondrial dynamics, ROS overproduction, and the release of proapoptotic proteins and other factors, resulting in cell death [30]. Mitochondrial quality control and dynamics are essential for regulating mitochondrial homeostasis. Excessive mitochondrial fragmentation with increased fission or impaired fusion is a hallmark of many degenerative diseases and contributes to their pathophysiology [31, 32]. It was also reported that hepatitis B virus induces mitochondrial fission, which contributes to mitochondrial dysfunction and cell injury [33]. The processes of mitochondrial dynamics are regulated by conserved dynamin-related GTPases, including the fission proteins DRP1, FIS1, and MFF and the fusion proteins MFN1, MFN2, and OPA1 [34]. Phosphorylation of DRP1 at Ser616 and translocation from the cytoplasm into mitochondria seem to be the key upstream events of mitochondrial fragmentation and dysfunction [35]. In the present study, we also found that selonsertib could effectively suppress DRP1 activation and mitochondrial translocation, rescue mitochondrial dysfunction and suppress mitochondrial oxidative stress in the liver tissue of ALF. Since JNK activation is thought to be critically controlled in the processes of DRP1-mediated fission, the protective role of selonsertib on ALF may occur through modulating mitochondrial dynamics by the JNK–DRP1 pathway.

Macrophages play a pivotal role in the pathophysiology of inflammatory diseases [36]. Several studies have proven that the danger of ALF is dependent on liver immune homeostasis and the inflammatory microenvironment, which is mainly modulated by macrophages [19, 37]. As LPS exposure is known to induce macrophage mitochondrial dysfunction in acute tissue injury [21], we then investigated the effect of selonsertib in protecting the mitochondrial function and dynamics of hepatic macrophages and also used LPS-primed macrophages as an in vitro model to determine this issue. We found that selonsertib pretreatment effectively prevented LPS-induced activation of JNK and DRP1 and mitochondrial dysfunction in primary hepatic macrophages and macrophagic cell line and also suppressed the release of the proinflammatory cytokines TNF-α and IL-1α, which are known as major cytokines causing liver injury and inflammation [38, 39]. By using mdivi-1, a selective inhibitor of DRP1, we further confirmed the role of DRP1 in mediating LPS-induced mitochondrial dysfunction in macrophages and LPS/GalN-induced fulminant liver injury and inflammation. These data indicate that DRP1 may be the key molecule contributing to the protective effect of selonsertib on ALF by affecting the mitochondrial function of macrophages. Although previous studies have mainly focused on the role of ASK1 in modulating cytotoxic stress-induced hepatocellular necrosis and apoptosis [16, 27, 40], our study showed that the therapeutic effect of ASK1 inhibition on ALF may also occur through protecting macrophage mitochondrial function.

## Conclusion

In summary, our data identified the specific ASK1 inhibitor selonsertib as a potential therapeutic drug for the early treatment of ALF by inhibiting JNK-mediated DRP1 mitochondrial translocation and then rescuing mitochondrial damage. In addition, our study confirmed the important role of mitochondrial function in the pathogenesis of ALF by determining the interaction between ASK1–JNK–DRP1 axis-mediated mitochondrial shape and the abnormal secretion of inflammatory cytokines in macrophages, suggesting that restoring mitochondrial homeostasis may also offer a novel strategy for ALF therapy development.

## Supplementary Information

**Additional file 1: Figure S1.** Selonsertib suppresses p38 activation in LPS/GalN-induced liver failure. (A)Western blot analysis and gray value assay on p-ASK1, ASK1, p-p38 and p38 levels in murine liver samples. Data are presented as mean ± SD (n = 6),**P < 0.01; NC, normal control; SEL, selonsertib (30 mg/kg). (B)Western blot analysis on p-p38 and p38 levels in murine liver samples at initial (0 h) and 0.5 h, 1 h, 2 h, 4 hand 6 h after LPS/GalN injection. Two from 6 samples were shown in the blots.

## Data Availability

All data and materials are available upon request.

## Abbreviations

ALF: Acute liver failure
ASK1: Apoptosis signal-regulating kinase 1
MAP3K: Mitogen-activated kinase kinase kinase
NASH: Non-alcoholic steatohepatitis
MDR: Multidrug resistance
LPS/GalN: Lipopolysaccharide and d-galactosamine
ALT: Alanine aminotransferase aspartate
AST: Aspartate aminotransferase
TBiL: Total bilirubin
ELISA: Enzyme-linked immunosorbent assay
TNF-α: Tumor necrosis factor-α
IL-1α: Interleukin-1α
CBA: Cytometric Bead Array
MCP-1: Monocyte chemoattractant protein-1
IFN-γ: Interferon-γ
mPTP: Mitochondrial permeability transition pore
Δψm: Mitochondrial membrane potential
DRP1: Dynamin-related protein 1
FIS1: Mitochondrial fission protein 1
MFF: Mitochondrial fission protein
MFN1: Mitofusin 1
MFN2: Mitofusin 2
OPA1: Optic atrophy 1
VDAC1: Voltage-dependent anion channel 1
