# Supplementary material for: Selonsertib, a potential drug for liver failure therapy by rescuing the mitochondrial dysfunction of macrophage via ASK1–JNK–DRP1 pathway
Source: Cell Biosci. 2021 Jan 7;11:9. doi: 10.1186/s13578-020-00525-w (PMC7792153; doi:10.1186/s13578-020-00525-w)
Supplement: Supplementary file 1 — Additional file 1: Figure S1. Selonsertib suppresses p38 activation in LPS/GalN-induced liver failure. (A)Western blot analysis and gray value assay on p-ASK1, ASK1, p-p38 and p38 levels in murine liver samples. Data are presented as mean ± SD (n = 6),**P < 0.01; NC, normal control; SEL, selonsertib (30 mg/kg). (B)Western blot analysis on p-p38 and p38 levels in murine liver samples at initial (0 h) and 0.5 h, 1 h, 2 h, 4 hand 6 h after LPS/GalN injection. Two from 6 samples were shown in the blots. [file 13578_2020_525_MOESM1_ESM.pdf]

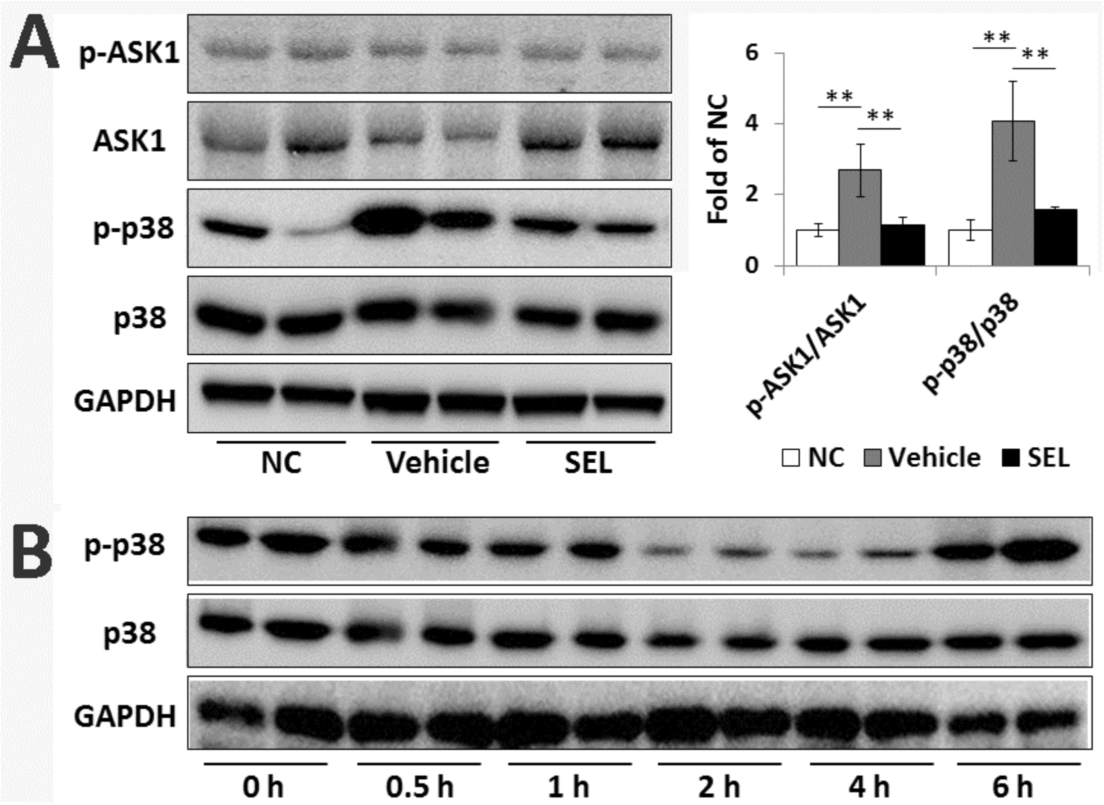

Figure S1. Selonsertib suppresses p38 activation in LPS/GalN-induced liver failure

(A) Western blot analysis and gray value assay on p-ASK1, ASK1, p-p38 and p38 levels in murine liver samples. Data are presented as mean ± SD (n=6), $^{**}P < 0.01$; NC, normal control; SEL, selonsertib (30 mg/kg). (B) Western blot analysis on p-p38 and p38 levels in murine liver samples at initial (0 h) and 0.5 h, 1 h, 2 h, 4 h, and 6h after LPS/GalN injection. Two from 6 samples were shown in the blots.
